# Notice to Subscribers

**Published:** 1898-09

**Authors:** 


### NOTICE TO SUBSCRIBERS.

On another page, under the head of *Correspondence,* will be found a query from one of our subscribers. Now, this is only the beginning of what we should like to make the most interesting part of our journal. We solicit from you such queries and answers as may be of interest to you, and the same will no doubt prove interesting to others. Many a physician has some original method of treatment which has proven invaluable to him in the treatment of certain diseases. Why not give these to your professional brethren? Give a short, succinct report of some interesting case, and the manner in which it was treated. We have long ago held that the attendance upon society meetings was invaluable to the physician simply for the reason that it allowed different expression of ideas upon the same subject, and this is always beneficial. Why not accomplish the same thing in a medical journal through correspondence? Your case is perhaps familiar to some other brother practitioner, and if you need aid he will be glad to give it. Let us hear from you, and let us make the "Correspondence Department" highly entertaining.

